# Transient Activation of Apomixis in Sexual Neotriploids May Retain Genomically Altered States and Enhance Polyploid Establishment

**DOI:** 10.3389/fpls.2018.00230

**Published:** 2018-02-26

**Authors:** Diego Hojsgaard

**Affiliations:** Department of Systematics, Biodiversity and Evolution of Plants, Albrecht-von-Haller Institute for Plant Sciences, Georg August University of Göttingen, Göttingen, Germany

**Keywords:** apomeiosis, euploid gametes, genomic resilience, genomic shock, meiosis, neopolyploids, parthenogenesis, triploid bridge

## Abstract

Polyploid genomes evolve and follow a series of dynamic transfigurations along with adaptation and speciation. The initial formation of a new polyploid individual within a diploid population usually involves a triploid bridge, a two-step mechanism of cell fusions between ubiquitous (reduced) and rare (unreduced) gametes. The primary fusion event creates an intermediate triploid individual with unbalanced genome sets, a situation of genomic-shock characterized by gene expression dysregulation, high dosage sensitivity, disturbed cell divisions, and physiological and reproductive attributes drastically altered. This near-sterile neotriploid must produce (even) eupolyploids through secondary fusion events to restore genome steadiness, meiotic balance, and fertility required for the demographic establishment of a nascent lineage. Natural conditions locate several difficulties to polyploid establishment, including the production of highly unbalanced and rarely unreduced (euploid) gametes, frequency-dependent disadvantages (minority cytotype exclusion), severe fitness loss, and ecological competition with diploid parents. Persistence and adaptation of neopolyploids depend upon genetic and phenotypic novelty coupled to joint selective forces that preserve shock-induced genomic changes (subgenome homeolog partitioning) and drive meiotic (reproductive) stabilization and ecological diversification. Thus, polyploid establishment through the triploid bridge is a feasible but not ubiquitous process that requires a number of low-probability events and singular circumstances. Yet, frequencies of polyploids suggest that polyploid establishment is a pervasive process. To explain this disparity, and supported in experimental evidence, I propose that situations like hybridization and ploidy-state transitions associated to genomic shock and substantial developmental alterations can transiently activate apomixis as a mechanism to halt genomic instability and cancel factors restraining neopolyploid’s sexual fertility, particularly in triploids. Apomixis –as a temporal alternative to sex– skip meiosis and syngamy, and thus can freeze genomic attributes, avoid unbalanced chromosomal segregation and increase the formation of unreduced euploid gametes, elude frequency-dependent reproductive disadvantages by parthenogenetic development of the embryo and permissive development of endosperm during seed formation, and increase the effective population size of the neopolyploid lineage favoring the formation rate of eupolyploids compared to aneuploids. The subsequent action of genome resilience mechanisms that alleviate transcriptomic shock and selection upon gene interactions might restore a stable meiosis and sexual fertility within few generations, as observed in synthetic polyploids. Alternatively, provided that resilience mechanisms fail, the neopolyploid might retain apomixis and hold genomically and transcriptionally altered states for many generations.

## Introduction

Whole-genome duplication studies have revealed hidden polyploid ancestries in many angiosperm lineages, confirming that spontaneous polyploid formation is a usual mode of speciation with wide consequences for plant adaptation, ecology, and evolution (Van de Peer et al., 2017). The number and distribution of polyploids species highlight both the ecological flexibility as the evolvability of polyploid genomes and its relevance for angiosperms diversity (Meyers and Levin, 2006; Soltis et al., 2015). During their evolutionary road, new polyploids are committed to adapt and survive to ecological constraints by going through a series of stages characterized by gradual genetic and genomic changes. Polyploids arise in a diploid parental population and must produce a group of mating partners to become locally established. If they manage to persist, they may participate in various speciation processes before going extinct. During this succession, the initial stage necessarily goes through a statistically odd situation accompanied by genomic and transcriptomic shocks that the new polyploid must overcome whereas it becomes demographically established. Persistence, adaptation, and expansion of *neo*polyploids depend upon genetic and phenotypic novelty coupled to the preservation of shock-induced genomic changes (like subgenome homeolog partitioning) under which joint selective forces drive meiotic (reproductive) stabilization and ecological diversification (e.g., Bomblies et al., 2015; Soltis et al., 2015). These processes operating immediately after polyploid formation are responsible for a gradual transfiguration of *neo*polyploids into *meso*polyploids, and later *paleo*polyploids via structural and functional genome reorganizations, including subfunctionalization and fractionation of duplicated sequences, and restoration of a diploid-like meiotic segregation (Ramsey and Schemske, 2002; Hegarty and Hiscock, 2008; Freeling et al., 2015; Hollister, 2015).

**FIGURE 1:**
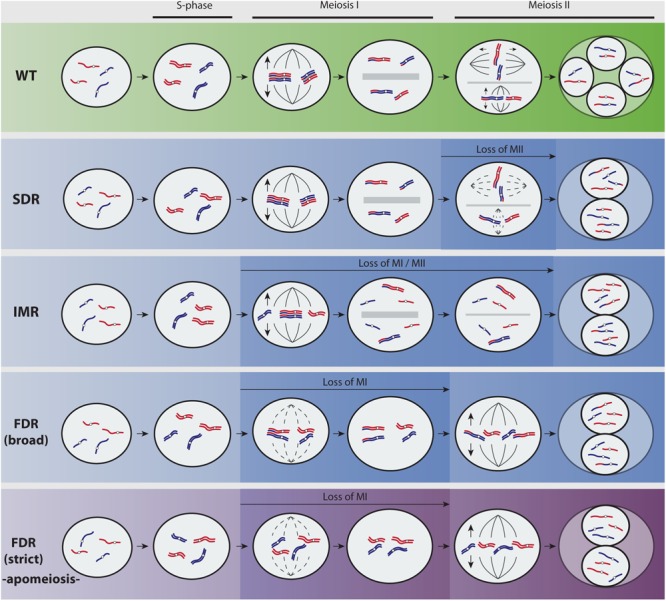
Overview of predominant mechanisms to unreduced gamete formation in angiosperms (adapted from Brownfield and Köhler, 2011; De Storme and Geelen, 2013). A hypothetical diploid plant with two chromosomes parentally differentiated (red and blue) is represented. Most flowering plants reproduce by means of seeds formed after syngamy of meiotically reduced male and female gametes (Wild Type, green pathway). Yet, meiosis fails at very low frequencies and a restitution division during first or second meiotic divisions (FDR and SDR, respectively) produces unreduced (2*n*) gametes with varied genetic constitutions (blue pathways show recombined parental heterozygosity and purple pathway retains parental heterozygosity). During SDR, meiosis I proceeds normally and pairing and recombination of chromosomes produce heterozygous 2*n* gametes. In particular cases, partial homeologous recognition leads to both bivalent and univalent formation that divides reductionally or equationally, respectively, and generates 2*n* gametes displaying functional omission of the first or second meiotic division among chromosomes (indeterminate meiotic restitution). The molecular and cytological basis for FDR and SDR are mainly associated to chromatid cohesion, chromosomal segregation, spindle orientation, cytokinesis defects, and cell cycle progression (Brownfield and Köhler, 2011; De Storme and Geelen, 2013). During FDR, meiosis I is lost through a fail of homeologous pairing due to asynapsis or desynapsis. Whereas certain level of recombination before restitution may be possible (FDR broad, blue pathway), the complete omission of synapsis, recombination, and bivalent formation (FDR strict, purple pathway) results in chromatid segregation and 2*n* gametes that are genotypically identical to the parent. Cytological processes leading to FDR-type of 2*n* nuclei are less frequently found in plants than those generating SDR-type of 2*n* nuclei (Bretagnolle and Thompson, 1995; Ramsey and Schemske, 1998; Brownfield and Köhler, 2011; De Storme and Geelen, 2013). This is most likely connected to the higher complexity and the evolutionary relevance of the first meiotic division, and to the fact that certain important alterations inducing 2*n* gametes are only achievable during the second meiotic division (e.g., parallel spindles). However, a complete omission of meiosis I has sporadically been observed in sexual plant species owing to cytological problems (De Storme and Geelen, 2013), but it can occur regularly in apomictic plants (called diplosporous apomixis; for details on types of apomixis, refer to Asker and Jerling, 1992) owing to a genetic background wherein such 2*n* gametes show a competence to develop the seed embryo without fertilization (by parthenogenesis). Hence, the new seedling fixes the genetic and epigenetic states allowing the recurrent formation of unreduced gametes over the next generations.

**FIGURE 2:**
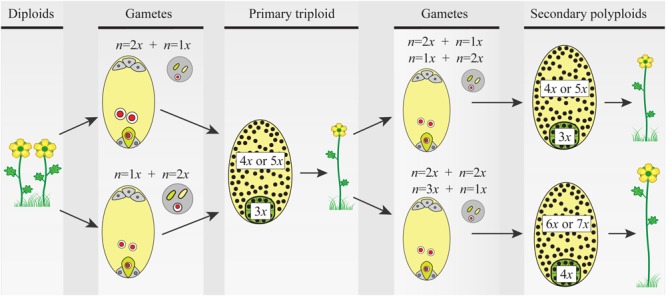
The triploid bridge mechanism. Two consecutive fusions of reduced–unreduced gametes are needed to produce a tetraploid individual from a diploid population. Unreduced gametes can be either female or male and contribute unequally to the embryo (2*m*:1*p* or 1*m*:2*p*, respectively) and the endosperm (4*m*:1*p* or 2*m*:2*p*, respectively) tissues of the seed. Thus, after the first fusion event, the formation of a triploid seed and growth of a seedling are constrained by phenomena like the triploid block, causing abnormal seed and plant development (details in the main text). Once a triploid plant arises in a diploid population, the formation of second-generation polyploid individuals is a precondition before the new polyploid lineage can become established. For simplicity, only euploid gametes are considered, but triploids produce an array of gametes with variable ploidy and new polyploids can be derived from a variety of pathways and mating options (see details in the main text and **Figure 3**). Here again, ploidy asymmetry between female and male gametes and chromosomally unbalanced gametes hamper seed and seedling developments reducing triploid’s fertility. In both steps of the triploid bridge, i.e., the formation of a primary triploid and secondary polyploids, the rate of formation of unreduced gametes, the minority cytotype disadvantages, and the effective size of the nascent population play a central role in the demographic establishment of new polyploids. Gametes: female gametophyte (oval) carrying two gametes, the egg cell (green), and the central cell (light yellow), and male gametophyte (circle) carrying two sperm nuclei (green and light yellow). Primary triploid and secondary polyploids: seeds (ovals) and ploidy of embryo (green) and endosperm (light yellow) tissues; the heterogeneous distribution of nuclei (black dots) illustrates developmental problems after parental genome contributions (details in the main text and **Figure 3**).

Of all phases of polyploid evolution, the formation and establishment stages are the most critical ones (Harlan and de Wet, 1975; Ramsey and Schemske, 1998). Cytological mechanisms responsible for the formation of unreduced gametes and new plant polyploids had been described (**Figure 1**). Most frequently, polyploidization involves a two-step mechanism of reduced–unreduced gamete fusions called “triploid bridge” (Ramsey and Schemske, 1998). During the first step, a regular haploid (*n*, reduced) gamete fuses to a rare diploid (2*n*, unreduced) gamete to form a triploid individual. Then, the primary triploid serves as a “bridge” between parental diploid(s) and produces a derivative tetraploid (**Figure 2**) which in turn must produce second-generation polyploid individuals through a variety of pathways (see, e.g., Ramsey and Schemske, 1998; De Storme and Geelen, 2013) to establish a group of homoploid mating partners. In nature, triploids are found sporadically, and the demographic establishment phase, i.e., the formation of the earliest assembly of even polyploid(s) starting from diploid ancestors, is the most elusive and less documented phase of a new-born polyploid. Current knowledge of factors governing polyploid establishment had been compiled from data gained through studies on the polyploid formation or from analyses of hitherto established polyploids coexisting with diploid parents (e.g., Ramsey and Schemske, 1998; Husband, 2004; Arrigo and Barker, 2012). Such studies offer robust evidence on key factors likely influencing neopolyploid establishment, which can be grouped in (1) rates of 2*n* gamete formation, (2) frequency-dependent mechanisms defining minority cytotype disadvantages (e.g., ecological niche differentiation, spatial heterogeneity, and reproductive isolation), (3) small effective population sizes (depending upon triploid bridge efficiency and/or existence of multiple primary polyploidization events), and (4) environmental changes that may further modulate 1–3 and increase polyploid establishment rates (Arrigo and Barker, 2012).

All these factors are interconnected and most likely have a variable incidence during the establishment phase on different plant systems, as suggested by previous studies revealing miscellaneous conditions under which new polyploid lineages may become established (reviewed in Ramsey and Ramsey, 2014; Soltis et al., 2014). Likewise, experimental evidence indicates that polyploid establishment through a triploid bridge is a feasible but not ubiquitous process that requires a number of apparently low-probability events and singular circumstances. Yet, the current numbers of polyploids suggest that polyploid establishment is a pervasive process (Meyers and Levin, 2006; Soltis et al., 2015). This discrepancy together with uncertainties observed in many cases of polyploids where singular conditions for their establishment are not met veil the early stages of polyploid establishment and indicate that there might be still unnoticed mechanisms, and/or the need of new perspectives. The purpose of this paper is to explore facts and provide a new view of the conditions under which a new triploid can promote (more efficiently) the establishment of polyploids.

As mentioned above, the establishment process requires the formation of one primary triploid individual. The creation of a small population of secondary triploids and/or tetraploids is central for the local establishment and a prerequisite to cope with ecological and reproductive competition to parental diploids (Fowler and Levin, 1984; Arrigo and Barker, 2012). Primary triploids might be formed multiple times in a diploid population (depending upon rates of unreduced gametes) and thus contribute to increasing the number of polyploid lineages in the long term. In the short term, however, simultaneous formation of primary triploids is not expected to have a great influence on the local establishment of secondary polyploids as recurrent polyploidization events are sporadic and independent (Soltis and Soltis, 1999; Soltis et al., 2012). Hence, a second primary polyploid will rarely be concurrent in the same spatial area, and even when a perennial habit may enhance the concurrence of primary polyploids, perenniality is more relevant when it comes to the formation of secondary polyploids mainly because triploids had low fitness and are short-lived. Nonetheless, there are numerous annual plants which are successful polyploids, including many crop species (Gustafsson, 1948; Ramsey and Ramsey, 2014).

In a similar analysis, since polyploidization is an *in situ* mechanism, ecological differentiation and reproductive isolation between parental diploids and their polyploid progeny are not immediate (e.g., Husband and Sabara, 2004; Slotte et al., 2008), and therefore these mechanisms lack drastic short-term effects on local establishment and cannot explain many cases of sympatric successful polyploids. Instead, habitat segregation by niche differentiation can avoid competitive exclusion by diploid ancestors and has been invoked as a primary mechanism facilitating polyploid establishment (Husband and Schemske, 2000; Levin, 2003) and fostering rapid adaptive divergence between chromosomal races (e.g., between tetraploids and hexaploids in *Achillea*; Levin, 2011; Ramsey, 2011). However, the niche shift hypothesis is not conclusive in all cases. Even when established polyploids are often reported to occupy physically harsh environments beyond the ecological tolerance of their diploid ancestors (e.g., Kirchheimer et al., 2016; Karunarathne et al., 2018), the selective processes that might promote niche divergence are not immediate, and experimental tests of niche differentiation among diploids and polyploids have delivered equivocal results in many cases (e.g., Baack and Stanton, 2005; Buggs and Pannell, 2007; Raabová et al., 2008).

The triploid bridge is likely the most influential mechanism to the local demographic establishment of secondary polyploids. The efficiency of the triploid bridge relies on triploid’s fertility, which is mainly driven by the formation of chromosomally balanced gametes and by interploid reproductive isolation (Khush, 1973; Ramsey and Schemske, 1998). Overall, for any mechanism to increase the triploid bridge efficiency, it must enhance the formation rate of new eupolyploid individuals in the short term, either from backcrosses with parental diploids or from secondary neopolyploids. Current modeling suggests that 2*n* gamete frequencies may explain the ploidy heterogeneity observed in nature (e.g., Husband, 2004; Oswald and Nuismer, 2011; Suda and Herben, 2013). However, for simplicity and feasibility, mathematical modeling requires the assumption of rather idealistic biological parameters, e.g., the existence of previously established tetraploids or cytotypes of higher ploidy (as persistent sources of unreduced-like gametes), high triploid fitness (high viability and/or fertility), the absence of aneuploid gamete formation in odd cytotypes, immediate reproductive isolation with diploid parental populations, or the absence of cytotype-frequency disadvantages. In spite all these are conceivable conditions, they are hardly met by neotriploids in nature.

## Triploid’s Fate: A Convergence of Natural Forces and Abnormal Genomic and Reproductive States

The appearance of new triploids and their functional role exhibit different constraints, being the formation of unreduced gametes the starting point. Different studies have shown that rates of 2*n* gamete production are variable, generally low (<0.5%, but see Ramsey and Schemske, 1998 for details on frequency of unreduced gametes in diploid hybrids and non-hybrids), and influenced by environmental factors (Harlan and de Wet, 1975; Ramsey and Schemske, 1998; Mason et al., 2011; De Storme and Geelen, 2013; Kreiner et al., 2017). Even though estimations indicate that new polyploids can be generated through a triploid bridge, they are largely based on results from experimental crossings and laboratory conditions, in many cases using agricultural or horticultural plants previously selected for their high production of gametes, and according to Ramsey and Schemske (1998, p. 490), they “may not be completely indicative of natural populations.”

Triploids are relatively common in diploid–tetraploid contact zones, as by-side products of interploid matings (e.g., Sabara et al., 2013; reviewed in Husband et al., 2013), and have relevant roles for the coexistence of cytotypes and gene flow between diploids and tetraploids (e.g., Husband, 2004; Kao, 2007). Elsewhere has been mentioned that contact zones contain diploids and polyploids in close geographic proximity and thus might mimic early stages of polyploid evolution. However, such assertion skips the very early stages when a primary triploid is formed in a diploid population without tetraploid individuals as an extra source of diploid gametes. In that particular evolutionary glimpse, the neotriploid must establish a number of second-generation (triploid and/or tetraploid) eupolyploids under dramatically unequal conditions.

Naturally occurring sexual triploid plants immersed in a diploid population are rarely found. The formation of a triploid seed by a diploid mother must first overcome some developmental issues. Unbalanced maternal and paternal genome contributions during fusion of gametes displaying ploidy asymmetry cause what is called the triploid block, a phenomenon characterized by malfunction of endosperm and aberrant seed development owing to altered gene dosage effects and imprinting of regulatory Polycomb Group MEDEA genes (Scott et al., 1998; Erilova et al., 2009; Köhler et al., 2010; Kradolfer et al., 2013; Lafon-Placette and Köhler, 2016). The triploid block has been issued as a mechanism for reproductive isolation and angiosperm speciation (Cooper and Brink, 1942; Gutierrez-Marcos et al., 2003; Lafon-Placette and Köhler, 2016). Whereas the triploid block can reinforce the isolation in diploid–tetraploid contact zones, it represents an impediment to the formation and fertility of neotriploids.

**FIGURE 3:**
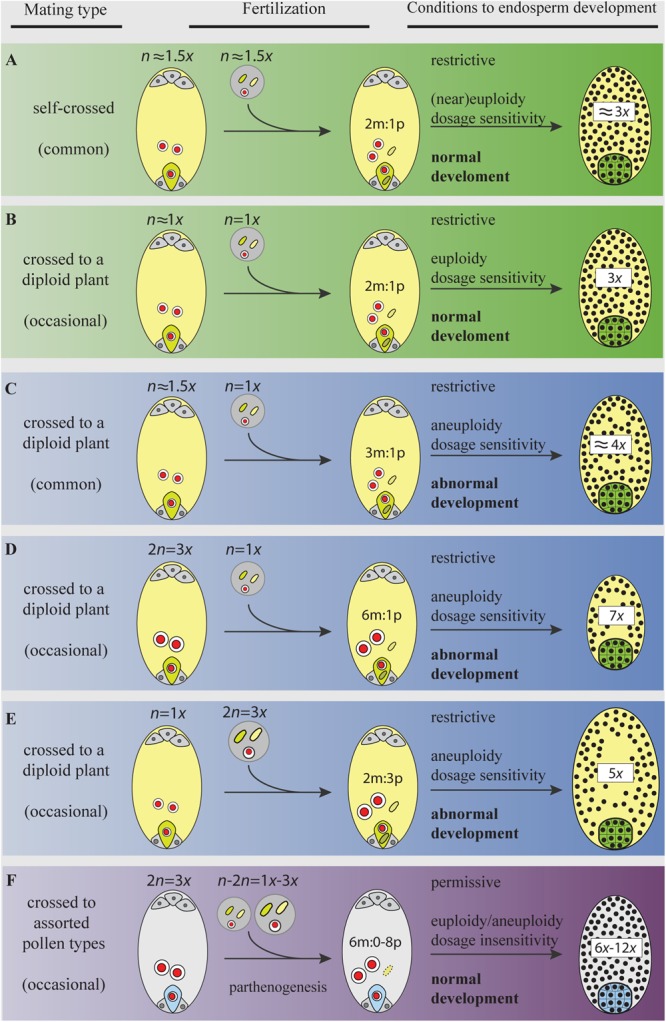
Gamete ploidy, mating options, and constraints to seed formation in a neotriploid under meiosis, syngamy, and parental genome dosage (sexual) requirements **(A–E)**, and under apomeiosis, parthenogenesis, and dosage insensitivity (apomixis) requirements **(F)**. Meiosis and sexual reproduction are under control of dozens of genes and epigenetic signals that regulate the normal progression through sporogenesis, gametogenesis, embryo-, and endosperm-genesis (Kawashima and Berger, 2014). Abnormal chromosome segregations in the triploid generate a swarm of aneuploid gametes. Only gametes with the same or similar ploidy will go through the restrictive 2*m*:1*p* genome dosage requirements of the endosperm primary cell and develop a functional seed **(A,B)** (Scott et al., 1998; Lafon-Placette and Köhler, 2016). Yet, aneuploid gametes having similar chromosome numbers (e.g., in **A**) may not share the same assortments of chromosomes and will face aberrant seed development. Distorted chromosome pairs disrupt gene expression and lead to developmental defects (Birchler and Veitia, 2009).*(In plants, an excess of maternal genomes decelerate endosperm mitosis, exhibit precocious cellularization, embryo arrest, and produce aberrantly small seeds **(C,D)**; a paternal excess accelerates mitosis, block cellularization, embryo arrest, and produce abnormally large seeds **(E)** (Scott et al., 1998; Lafon-Placette and Köhler, 2016). When parental gametes are chromosomally unbalanced (in **C,D**), the development of embryos will face genetic and phenotypic costs of aneuploidy. The activation of apomeiosis can increase the formation of euploid female gametes, but more important, when coupled to parthenogenesis, it shields the euploid embryo from merging to aneuploid gametes in ineffective matings. In addition, apomicts are insensitive to shifts in dosages of parental genomes involved in endosperm formation (Quarin, 1999; Talent and Dickinson, 2007), a condition for which the underlying molecular mechanisms remain unexplored. Bypassing the requirement for a 2*m*:1*p* genome contributions to the endosperm is particularly relevant in triploids and odd polyploids being exposed to a high proportion of aneuploid or heteroploid gametes. Thus, for a neotriploid, the activation of apomixis represents a permissive environment to seed formation, where the two maternal nuclei in the central cell can fuse to male gametes of assorted-ploidy (from self-pollen or pollen from adjacent diploids), and yet develop a functional endosperm and keep the euploid condition of the embryo **(F)**. Making female gametes receptive to a wider range of male ploidies increases the number of available male gametes from the gamete pool in interploid crosses. In triploid plants (even in diploid ones), the emergence of apomeiotic female gametophytes will have an advantage over meiotic ones by skipping developmental pressures during interploid pollinations. Female and male gametophytes, gametes, and seeds are represented following the descriptions in **Figure 2**. The green egg cell (and embryo) and the light yellow central cell (and endosperm) in **(A–E)** represent gametes (and seeds) produced after meiosis (and syngamy); the light blue egg cell (and embryo) and gray central cell (and endosperm) in **(F)** represent apomeiotic and parthenogenetic female gametes (and seed).)*

Once a first triploid plant is rooted, it must then overcome a reproductive bottleneck caused by their genetic condition. Triploids exhibit abnormal meiosis, the formation of chromosomally unbalanced gametes, and largely reduced fertility (see, e.g., Abbott and Lowe, 2004; Duszynska et al., 2013). Since a primary neotriploid will necessarily be embedded in a diploid parental population lacking other polyploid mates, they will suffer a strong negative frequency-dependent selection via ineffectual matings with diploid parents (Levin, 1975) (**Figure 3**). While surrounding diploids produce abundant haploid gametes (1*x*), triploids produce lower numbers of gametes, mostly aneuploid, exhibiting an array of chromosomal combinations (from ∼1*x* to ∼2*x*) (Ramsey and Schemske, 1998; Henry et al., 2005; Martínez et al., 2007; Duszynska et al., 2013). In addition, a ratio of two maternal genomes to one paternal genome (2*m*: 1*p*) is deterrent for normal endosperm development which allocates a strong selection against aneuploid gametes in favor of euploid gametes (Ramsey and Schemske, 1998, 2002; Henry et al., 2005; Lafon-Placette and Köhler, 2016; but see below). Thus, successful seed formation generally occurs when both gametes match their chromosome numbers. Different experimental studies show high levels of ineffectual gamete mattings in 2*x* ↔ 3*x* crosses that depress triploid fitness and triploid fertility, whereas probabilities of formation of new zygotes of ploidy 2*x*+*y* (where *y* represents supernumerary chromosomes in gametes with 1*x*+*y* chromosomes from the triploid parent) increase as *y* approaches 0 (zero) (e.g., McClintock, 1928; Henry et al., 2007; Martínez et al., 2007; Rounsaville et al., 2011).

Still, under laboratory conditions, triploids may produce tetraploids. After several generations of obligated selfing, Henry et al. (2005) found a gradual shift toward euploid progenies that finally resolved the starting triploid’s aneuploid swarm into two groups, one of near diploids–eudiploids and another one of near tetraploid–eutetraploids. The authors found evidence that a single locus they called *sensitive dosage imbalance* (SDI) exhibited segregation distortion in the subgroup of near tetraploids only, suggesting that SDI promotes survival of extreme aneuploids by buffering the effects of dosage imbalance (Henry et al., 2007). The study indicates that triploids can bridge toward tetraploids through several generations of aneuploidy. Experimental conditions are far to resemble the natural environment, and many selective forces that may act upon triploids’ aneuploid and physiologically deficient offspring during first generations are probably abolished in the laboratory. Nevertheless, recently established and resynthesized eupolyploids depict massive chromosomal variation and hidden aneuploidy through reciprocal chromosomal compensation, suggesting that first neoallopolyploids may take advantage of these chromosomal mechanisms as a source of genetic variation, and experience periods of aneuploidy and rearrangements before stabilizing their karyotype and genomic attributes (Xiong et al., 2011; Chester et al., 2012; but see Zhang et al., 2013 for a case of apparent transgenerationally persistent aneuploidy). In these cases, selection for euploidy is explained by the gene balance hypothesis, as products of genes in aneuploid chromosomes may cause stoichiometric imbalances of macromolecular complexes and have superior effects than whole-genome changes (Birchler and Veitia, 2012). The rare occurrence of aneuploids on natural populations (i.e., nullisomy, monosomy, trisomy, etc.) in the 2*x–*3*x* (and 3*x–*4*x*) ploidy range suggests that if selection for euploidy via gene–dosage balance and/or SDI-like effects is a common process among angiosperms, it might only require a few generations.

In nature, the selection for euploid offspring must occur during the first generations after triploid formation, and hence it must be concurrent with the state of genomic shock observed after polyploidization and/or hybridization events. Such state refers to genomic instabilities caused by rapid and extensive genomic reorganization, including chromosome rearrangements, gene loss, and epigenetic alterations with dramatic changes in gene expression and phenotypic instabilities (e.g., Comai et al., 2000; Adams and Wendel, 2005; Stupar et al., 2007; Madlung and Wendel, 2013). During this initial period of genomic stress, the success of the new polyploid is hampered by plant lethality or reproductive sterility (Mayrose et al., 2011; De Storme and Mason, 2014). If the new polyploid succeeds in attaining the demographic establishment of a cohort of eupolyploid mating partners, varied post-polyploidization structural genomic changes can be retained in new lineages (Mavrodiev et al., 2015) and contribute to increase polyploid phenotypic flexibility, ecological niche differentiation, and adaptation to new environments (e.g., te Beest et al., 2012).

Overall, triploids represent a transient evolutionary state characterized by karyotypic, genomic, and reproductive instability, and a short-lived existence. In natural conditions, diploid parents drive seedling ploidies, and abnormal meiosis, dilution of exceptional 2*n* gametes in the total gamete pool, endosperm failure, and seed abortion hamper the chances of a primary triploid to produce a group of (eu)triploid or (eu)tetraploid individuals. Only three exceptional cases of semi-fertile sexual triploids are known. In those triploids, unusual meiotic systems maintain a permanent odd polyploidy through asymmetric chromosomal and genome segregations into female and male gametes [*Leucopogon juniperinus* R. Br. (Smith-White, 1948); *Cardamine × insueta* Urbanska-Worytkiewicz (Urbanska-Worytkiewicz, 1977; Grant, 1981); and *Andropogon ternatus* (Spreng.) Nees (Norrmann and Quarin, 1987)]. All other known long-lived triploids maintain their fertility by skipping meiosis and sexuality and reproducing through apomixis, a mechanism that produces unreduced female gametes and seed embryos without requiring a male contribution [some examples are *Erigeron annuus* Pers. (Noyes, 2000); *Eupatorium sessilifolium* L. (Sullivan, 1976; Grubbs et al., 2009); *Hieracium alpinum* L. (Mráz et al., 2009); *Paspalum quadrifarium* Lam. (Quarín and Lombardo, 1986); and *Taraxacum officinale* F. H. Wigg. (van Dijk, 2003)]. The main adaptive advantage of apomixis is the restoration of fertility in sexually sterile individuals (Darlington, 1939; de Wet and Stalker, 1974). The emergence of mechanisms dodging meiotic abnormalities and imposing interploid barriers to the surrounding male diploid mates upholds fertility and has strong consequences on the functional output of triploid plants. But could the efficiency of a primary triploid working as a bridge for the establishment of new even polyploid individuals rely on the temporary activation of mechanisms with a transient role? Could a newly arisen triploid activate an escape of sexuality to enhance the formation of secondary eupolyploids?

## Might Triploids Transiently Activate Apomixis and Boost the Bridge Efficiency?

Apomixis is a complex process and a rare condition where a plant has fixed a genomic configuration that skips sexual programs and develops clonal seeds. Apomictic individuals show an enduring capacity to bypass female meiosis (i.e., apomeiosis) thereby producing a high proportion of unreduced megagametophytes, wherein egg cells develop an embryo by parthenogenesis (i.e., fertilization-free), and the required two maternal-to-one paternal genome dosage (2*m*:1*p*) in the endosperm is relaxed (the endosperm can develop under sperm-free conditions or with male contributions of varied ploidy) (**Figure 3**). Genomic and phenotypic features in apomicts are defined by multiple factors. Apomictic plants show a block of recombination in reproductive-related genome regions which are inherited as a single dominant factor, exhibited interrupted gene sequences, modified epigenetic signals, and altered gene silencing pathways and gene expression levels (Ozias-Akins and van Dijk, 2007; Garcia-Aguilar et al., 2010; Olmedo-Monfil et al., 2010; Polegri et al., 2010; Sharbel et al., 2010; Verhoeven et al., 2010; Hojsgaard et al., 2011; Podio et al., 2014; Schmidt et al., 2014). Additionally, compared to the sexual program, apomicts display temporal–spatial developmental asynchrony, incomplete penetrance, and variable expressivity environmentally modulated (Grimanelli et al., 2003; Ozias-Akins and van Dijk, 2007; Hojsgaard et al., 2013, 2014a; Schmidt et al., 2014). Since the male does not contribute to the development of the plant embryo in apomictic seeds, genetic and genomic attributes are transmitted intact to the offspring (Ozias-Akins and van Dijk, 2007; Sailer et al., 2016). However, due to incomplete penetrance, apomictic plants can produce some sexual seeds (residual sexuality) thus allowing partial reshuffling of genomic regions not linked to the character with long-term evolutionary consequences (e.g., van Dijk et al., 2009; Hojsgaard and Hörandl, 2015a). Numerous genes have been identified to play functional roles in apomictic development on diverse apomictic plant systems (Albertini et al., 2005; Corral et al., 2013; Siena et al., 2014; Conner et al., 2015; Pellegrini, 2016; Worthington et al., 2016), but information on gene regulatory programs underlying apomixis is limited. The *ASGR–BBML* gene family seemingly had a shared function on parthenogenesis among different apomictic species (e.g., Conner et al., 2015, 2017; Worthington et al., 2016), and mutations on important meiotic genes like *DYAD/SWITCH1*, or the MiMe triple mutant can transmute meiosis into mitosis in sexual plants (Ravi et al., 2008; d’Erfurth et al., 2009). However, a common molecular basis for apomixis has not yet been clarified. Altogether, apomeiosis and parthenogenesis had different genetic and epigenetic basis (e.g., Hand and Koltunow, 2014; Podio et al., 2014) and can occur uncoupled. Even though the precise connection between both elements at the molecular level is still unknown, an evolutionary perspective makes clear that their separate expression would be untenable in the long term, creating organisms genetically and physiologically unviable, with deleterious upgrading or downgrading ploidies (Asker and Jerling, 1992). Apomictic plants *per se* are relatively rare in nature, occurring in one or more species in 293 angiosperm genera (2.3% of all genera; Hojsgaard et al., 2014b), and almost exclusively linked to odd and even polyploids (Asker and Jerling, 1992; Carman, 1997). Congeneric diploids show a tendency to produce low proportions of apomeiotic female gametes (e.g., Norrmann et al., 1989; Naumova et al., 1999; Hojsgaard et al., 2008). However, functional apomixis is not stable at diploid level (Bicknell, 1997; Siena et al., 2008; Delgado et al., 2014; Schinkel et al., 2016; but see dihaploids in *Erigeron*, Noyes and Wagner, 2014) and is only sporadically associated to persistent medium-to-high seed set and fertility in diploid cytotypes [*Boechera holboellii* (Hornem.) Á. Löve and D. Löve (Boecher, 1951; Kantama et al., 2007)].

Most reproductive biology studies are based on well-established (even) polyploids, and exhaustive evaluations on reproductive development of newly formed polyploids (particularly triploids) are scarce. Recent studies comparing the reproductive development of first-generation diploid and triploid synthetic hybrids from homo- and heteroploid crosses between sexual parents in the *Ranunculus auricomus* aggregate showed that female and male gamete developments are altered, unstable, and severely restrict the sexual pathway to seed formation in the newly formed hybrids (Hojsgaard et al., 2014a). At the same time, meiotic instabilities were accompanied with the appearance of low rates of apomeiosis in diploids and triploids, which is partly linked to parthenogenesis in triploids. Thus, neotriploids were able to produce clonal seeds via the formation of apomeiotically unreduced parthenogenetic female gametophytes, but not all apomeiotically derived egg cells developed into a zygote via parthenogenesis, and were fertilized to produce a seed embryo with an increase of the maternal ploidy [2*n*(3*x*) + *n*(*x*)] (Hojsgaard et al., 2014a). So, synthetic F_1_ neotriploid plants were able to bypass the highly disturbed meiosis and produce low rates of eupolyploids (both triploid and tetraploid cytotypes) via apomixis, besides the expected aneuploid sexual progeny. The 2*n* + *n* pathway of seed formation is widely known (as B_III_ hybrids; Rutishauser, 1948) and commonly found in different apomictic systems (e.g., Harlan and de Wet, 1975; Martínez et al., 1994; Burson et al., 2002; Ramsey, 2007; Krahulcová et al., 2011; Schinkel et al., 2016). However, the activation of apomixis elements in sexual derived crosses opens the question if this could be a transient but recurrent strategy used by odd polyploids and plants under situations of genomic stress to skip disturbed meiosis and reduced fertility. Its occurrence in natural sexual hybrids might have been overlooked just because its identification requires the use of specific methodological approaches, target (triploid) plants had a short-term life, because of the lack of sufficient material and/or difficulties to assess their reproductive biology.

The independent appearance of apomeiosis and parthenogenesis, or functional apomixis, might temporally alleviate genomic instability and cancel those factors restraining neopolyploid sexual fertility mentioned above. Mathematical modeling shows that parthenogenesis can facilitate the polyploidization process even under low polyploid fitness and severe triploid block (Yamauchi et al., 2004). The transient activation of apomeiosis and parthenogenesis at first may not change the triploid fitness (measured as seed set) but can enhance the triploid bridge efficiency during formation of secondary polyploids. First, apomixis might freeze the genomic structure of the neopolyploid bypassing meiosis (meiotic recombination and gametic assortment), and consequently shift the proportion of unreduced (euploid) gametes. Even at low proportions, apomeiosis will skip problems of chromosome pairing and segregation during meiosis and increase the number of euploid female gametes in comparison to an obligate sexual sib. Second, apomicts would elude frequency-dependent reproductive disadvantages of the minority cytotype by the pollen-independent parthenogenetic development of the egg cell into a clonal embryo, while the central cell would develop sperm-free or accept variable-ploidy sperms under endosperm-permissive conditions (see **Figure 3**). Thus, while sexual neopolyploids (especially triploids) being the minority cytotype are subject to greater ineffective (intercytotype) pollinations and consequent exclusion in local plant populations (Levin, 1975), parthenogenetic ovules are refractory to ineffectual pollinations and can elude such constraints. Third, the mentioned conditions favor the formation of eupolyploids (triploids and tetraploids, in the presence of both paternal haploid and aneuploid sperms) through seeds formed via apomeiosis + syngamy and apomeiosis + parthenogenesis; consequently, compared to an exclusively sexual system, such conditions accelerate the formation of polyploid individuals who contribute offspring to the next generation (i.e., the effective population size). Fourth, since rates of unreduced gametes (and so the expression of functional apomixis) are environmentally influenced (Quarin, 1986; De Storme and Geelen, 2011; Mason et al., 2011; Klatt et al., 2016), abiotic stress situations stimulate meiosis failure, and may well-stimulate apomeiosis, parthenogenesis, and with it a more efficient formation of secondary polyploids and the chances of survival of the polyploid lineage. The question remains how environmental changes would differentially affect speciation and extinction rates of diploids and polyploids (Arrigo and Barker, 2012, but see Freeling, 2017 and the discussion below).

## On Prevalent Diploids, Rare Triploids, Polyploid Establishment, and Natural Selection

Meiotic anomalies are central in the synthesis of new polyploids (Carputo et al., 2003; De Storme and Geelen, 2013). The formation of a new triploid from diploid parents represents an initial unstable state with a wide range of physiological, cytological, reproductive, genetic, epigenetic, and genomic alterations. Genomic instability and transcriptional shock during first generations post-hybridization have been identified in flowers of sexual plants (Hegarty et al., 2006). Altered gene expression changes (e.g., homeologous expression bias or transgressive upregulation) and widespread epigenetic repatterning are widely known to be caused by homo- and heteroploid hybridizations (Madlung and Wendel, 2013), and can be ameliorated by an increase in ploidy (e.g., through genome doubling; Comai, 2005; Hegarty et al., 2006). The resolution of such state involves several rounds of selection and the rise to an even-ploidy condition needed to recover a diploid-like pairing behavior, balanced chromosomal segregations, epigenetic reprogramming, and stabilized developmental and metabolic pathways (Soltis et al., 2012; Hegarty et al., 2013; Yant et al., 2013). Achieving these features is crucial for the new polyploid to restore sexual fertility and reproductive stability needed for to persist.

In natural and evolutionarily established sexual systems, meiosis and sexual fertility were functionally restored and stabilized by selection to avoid aneuploid gamete formation after auto- and alloploid establishments (Yant et al., 2013; Henry et al., 2014; Hollister, 2015) and it seems to be the pathway followed by sexual neopolyploid plants during the reinstatement of a diploid-like chromosomal segregation (Ramsey and Schemske, 2002; Madlung and Wendel, 2013; Bomblies et al., 2015; Hollister, 2015).

On the contrary, in natural established apomictic systems, meiosis and sexual fertility remain highly compromised, while medium-to-high levels of asexual seeds are formed (Asker and Jerling, 1992; Hojsgaard et al., 2013). Transcriptional profiling showed extensively altered patterns of gene expression on several regulatory pathways (e.g., cell cycle control, hormonal pathways, and epigenetic regulation; Grimanelli, 2012; Schmidt et al., 2014) of apomictic ovules compared to sexual ones. This massive gene dysregulation correlates to developmental asynchrony and switches from sexual to apomeiotic pathways, as observed in mature apomictic auto- and allopolyploid lineages as well as in F_1_ synthetic allopolyploid hybrids (Grimanelli et al., 2003; Polegri et al., 2010; Sharbel et al., 2010; Pellino et al., 2013; Hojsgaard et al., 2014a; Podio et al., 2014). However, little is known about the moment of emergence and establishment of apomixis in a new lineage. Yet, some clues come from the case study in *Ranunculus auricomus.* While natural 80,000 yo allopolyploids produce high rates of apomictic *vs.* sexual offspring (and traces of uncoupling events between apomeiosis and parthenogenesis), first-generation hybrids involving the same putative sexual parental species showed a contrasting pattern of low rates of functional apomixis compared to sexual progeny (and proportionally high percentage of uncoupling between apomeiosis and parthenogenesis) (Pellino et al., 2013; Hojsgaard et al., 2014a). Phylogeny, cytology, developmental features, gene expression profiling, and allelic diversity of extant natural apomicts and sexual ancestors support the observation that diversifying selection had acted onto meiotic and gametogenesis genes after formation of allopolyploid *Ranunculus* lineages to stabilize apomictic processes while retaining low rates of functional sexuality, altered gene expression, and signatures of past reproductive developmental instabilities (Pellino et al., 2013; Hojsgaard et al., 2014a).

These observations suggest that genome resilience in sexual neopolyploids can alleviate the genomic and transcriptomic shock caused by the initial hybridization and/or polyploidization events and restore genomic stability, balanced meiosis, and sexual fertility after selection upon gene interactions (Buggs et al., 2011; Hollister et al., 2012; Henry et al., 2014; Bomblies et al., 2015). In contrast, apomictic lineages fix a genomic configuration that lack of such genome resilience by retaining a genomic and transcriptional state alike that observed in recent polyploids, together with an abnormal meiotic program and low levels of functional sexuality. Late after the initial event, selection and residual sexuality may fine-tune the original transcriptomic shock toward more adaptive genomic configurations, both to respond to environmental stressors and to attain higher fertility through apomictic seeds. A situation like this is observed in clone members of a young *Taraxacum officinale* lineage exhibiting different evolutionary histories and functional divergence in metabolic pathways and heritable gene expression differences in response to biotic and abiotic stress (Ferreira de Carvalho et al., 2016).

**FIGURE 4:**
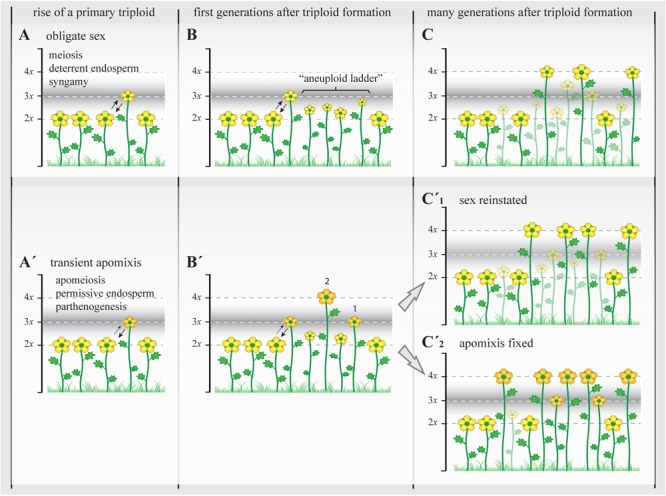
The triploid bridge efficiency and the establishment of a new polyploid lineage. Triploids represent a genomically and reproductively unstable temporary state with highly reduced fertility. In sexual triploids **(A)**, the formation of meiotically unbalanced gametes and the infrequent occurrence of unreduced (euploid) gametes lead to the creation of a variety of aneuploids, a “ladder” of physiologically unstable and ecologically subadapted cytotypes **(B)**, and therefore the establishment of even polyploids can take many generations and repeated polyploidization events. During this time, most subadapted aneuploid cytotypes will become extinct (transparent plants; stable triploid populations are rare among sexual angiosperms) and the new even polyploids **(C)** will carry many of structural genome reorganizations acquired during first generations before selection can re-stabilize meiosis and reinstate highly fertile genotypes (Yant et al., 2013; Hollister, 2015). When the initial genomic shock is associated with genetic and epigenetic changes that promote the appearance of apomixis **(A’)**, apomeiosis will promote the regular formation of unreduced gametes with or without parthenogenetic competence, leading to the formation of eutriploid and eutetraploid cytotypes in the first and subsequent generations after triploid formation (**B’**, 1 and 2, respectively) (Hojsgaard et al., 2014a). At this point, physiological homeostasis, genomic resilience, and epigenetic resetting could **(C’_1_)** gradually reinstate sexuality and lead to the formation of polyploid complexes with dissimilar genetic and genomic consequences as in **(C)** (though transient apomeiosis in triploids is expected to avoid structural genome reorganizations) that may promote more steady phenotypes and foster demographic establishment, or **(C’_2_)** it may gradually stabilize the genetic and epigenetic states that triggered apomixis and lead to the formation of agamic complexes (stable triploid populations are common among apomictic angiosperms). In both cases, i.e., with or without the appearance of apomixis, only the female side is considered as male gametes normally do not bypass meiosis in apomictic plants and therefore are chromosomally unbalanced in triploids and show similar ploidy outcomes to those observed in **A–C**. Yellow flowers represent sexual reproducing individuals; orange flowers represent individuals with apomixis. Different flower sizes illustrate relative fertility and adaptability among plants.

Under this view, it is simple to hypothesize the formation of a primary triploid holding an unstable genetic–epigenetic state wherein the provisional formation of apomeiotic unreduced gametes may be instated as an alternative to partially overcome meiotic abnormalities and fertility costs in the new odd polyploid, enhancing the establishment of secondary eupolyploids. If the transient formation of an unreduced gamete is caused by acquisition of genetic and/or epigenetic attributes that the cell cannot revert immediately (for example, a block of recombination and/or fail of DNA repairing mechanisms, features known to be associated with apomixis), that particular genomic configuration will become fixed in the new zygote. Such features associated with apomeiosis and parthenogenesis will be expressed at variable levels following the extent of sequences alteration and their interaction with trigger and modulating signals (either genetic and/or environmental). Then, uncoupling of apomeiosis and parthenogenesis (partial apomixis) during first generations after the emergence of the primary triploid will enhance triploid-to-tetraploid ploidy shifts as specified in the previous section. In the short term, such mechanisms will contribute to the formation of a group of secondary polyploids. In the medium- to long-term, chromosomal assortment in those neotetraploids and the resilience of the genome will contribute with two possible outcomes: (1) the restoration of a stable meiosis and sexual fertility through mechanisms that ameliorate genomic restructuring and repatterning, and alleviate the transcriptomic shock in the new mating group of even polyploids or (2) the fixation of a disturbed genomic configuration and the gradual selection toward higher levels of functional apomixis (apomeiosis + parthenogenetic competence) as an alternative to provide reproductive assurance (**Figure 4**). This view is also supported by the finding of rare triploids in geographically isolated natural populations of *Paspalum simplex* Morong exhibiting alternative reproductive states (one triploid reproduces by sexuality, the other one by apomixis; Urbani et al., 2002).

While the first outcome was already known for sexual polyploid plants, its connection to a conceivable transient activation of alternatives mechanisms to sex – previously unnoticed in primary neopolyploids – should not be compulsory. However, the possibility of alternate outcomes during the triploid bridge would benefit the explanation of polyploid establishment rates without excluding other accepted mechanisms as well as the lasting genomic and developmental alterations observed in apomicts. The low proportion of apomicts among angiosperms (despite advantages of asexuality) can be considered a simple signature left by a fail of the observed post-polyploidization resilience mechanisms that act upon neopolyploids to restore sexuality and stabilize the genome.

Evolutionary patterns on the Angiosperm phylogeny depict WGDs clustered around periods of climate change and unstable environments, indicating that polyploidization has a long-term role helping organisms to escape extinction (Van de Peer et al., 2017). To explain how polyploids managed to survive periods of mass extinction, Freeling (2017) proposed the “polyploidy as a spandrel of asexuality” hypothesis, according to which plant polyploids survive periods of unfavorable conditions by reproducing asexually and shielding reproductive meristems from environmental stress. Holding a time of asexual multiplication would relief the new polyploid of major problems of sexual reproduction, would upload mutations and benefit diploidization from an environment of relaxed selection, and could explain the “lag” in adaptive radiation observed post-polyploidization events (Schranz et al., 2012). The transient activation of apomixis fits Freeling’s hypothesis, and provides a more accurate frame of mechanisms and short-term advantages for the establishment of neopolyploids.

## Evolutionary Outlook

Polyploidy usually results in an evolutionary “dead end”. Polyploid plants reproducing through apomixis were traditionally considered as closed systems, losing genetic variability and evolvability, unable to provide genetic and phenotypic novelty, and therefore with no or little role in evolution (Stebbins, 1941). However, shifts to asexuality might provide polyploids with the evolutionary potential to overcome internal and external stressors. Former researchers noted that the occurrence of facultative and partial apomixis may function as an equilibrium system in evolution, providing a chance to propagate highly fitted genotypes and release variability during short periods of recombination in response to environmental challenges (Clausen, 1954). Now we know that apomictic lineages are genetically as diverse as their sexual relatives (e.g., Paun et al., 2006). Plants with facultative apomixis can fix genotypes exhibiting fittest gene combinations and concurrently exploit the advantages of residual sexuality to create new recombinant genotypes and elude negative effects like the accumulation of deleterious mutations (Muller’s ratchet), thus extending lifespan and speciation opportunities (Hojsgaard and Hörandl, 2015a,b). In recent years, the paradigm of apomixis as a closed system shifted toward one in which apomixis is a springboard for diversification, a transitional-phase facilitating neo- to paleopolyploid evolution within agamic complexes by reversals to sex (Carman, 1997; Hojsgaard et al., 2014b; Hojsgaard and Hörandl, 2015b; Hojsgaard et al., 2015). Additionally, by “hiding out” through asexual reproduction, polyploids could have better chances to survive during unfavorable conditions before re-establishing meiosis and sexual reproduction (Freeling, 2017). The present hypothesis of transient activation of apomixis during the formation of primary triploids and polyploid demographic establishment is consistent with this theoretical frame and adds mechanistic information on how elements alternative to meiosis and sexuality present in natural populations could enhance the triploid bridge, and the formation and demographic establishment of eupolyploids during the initial disruptive stages of a polyploid lineage. Understanding the origin of apomixis and the long-term consequences of divergent and/or stabilizing selection on meiotic and meiotic-related genes can help us to disentangle intricate phylogenetic patterns in polyploids and the role of apomixis within the evolutionary game.

## Author Contributions

DH conceived the idea, formulated the hypothesis, and wrote the manuscript.

## Conflict of Interest Statement

The author declares that the research was conducted in the absence of any commercial or financial relationships that could be construed as a potential conflict of interest.
